# In Medical and Surgical Practice

**Published:** 1920-08-14

**Authors:** 


### DIATHERMY.

### In Medical and Surgical Practice.

Our attention is drawn to this subject by two circumstances. The first is the appearance of another unit of the "Modern Methods of Treatment" series,* devoted on this occasion to the subject of Diathermy. The second is that for the moment anyone can legally give electric treatment without training or qualification. Whereas in actual fact, if certain dangers are to be avoided and beneficial results to be obtained, the employment of electrical therapeutic agencies should certainly be limited to those who have a certified working knowledge of their effects in diseased conditions. Also, a very considerable technical knowledge of electrical apparatus is essential, and yet at present anyone who possesses a dry battery can perform so-called treatment on a patient. The medical mind will at once appreciate what a deplorable state of affairs is often produced, and it is with the object of further illustrating the fact that we summarise certain sections of Dr. Claude Saberton's new book, as mentioned above.

### The Diathermic Principle.

Probably the best definition of diathermy in medical practice describes it as "a form of thermotherapy which utilises electrical energy for the production of thermal effects in the depth of the tissues." More technically one might say that diathermy is the high-frequency current under another name, and this leads to a brief explanation of how we are able to apply a really high-tension current to human living tissues at all. It is well known that electrical currents heat conductors through which they pass, but it is impossible to raise the internal temperature of the body by the ordinary *continuous* current on account of the pain produced by strong applications. The same reason prevents the use of the *faradic* current for this purpose; and this current also produces violent muscular contractions. In order to raise the internal temperature of the body we must employ currents which reverse their direction many thousand times per second, *i.e.*, *high-frequency* currents. In diathermy we are employing these heating effects, for the heat is generated in the tissues traversed by the current owing to the resistance they offer to the passage of a current through them.

Between the action of continuous and alternating currents in electrical conductors there are certain very marked differences. The first is that ordinary metallic wires or moistened fabrics which offer a relatively slight opposition to the continuous current, offer an immense resistance to the passage of high-frequency oscillations. Apropos of this phenomenon Professor J. A. Fleming writes: "If the difference of potential between the ends of a conductor is established with great suddenness, the resulting electric flow is less and less determined by what may be the true resistance and more and more by the inductance of the conductor. We have a mechanical analogy in the case of impulses or sudden blows given to heavy bodies, which well illustrates how strikingly force phenomena may be altered when for steadily and slowly varying forces we substitute exceedingly brief impulses or blows. . . . . The inductance of conductors introduces a series of phenomena which are the electric analogues of mechanical experiment. A conductor of sensible inductance can no more have a current of finite magnitude created in it instantaneously than a body of sensible mass can have a finite velocity instantaneously given to it. In both cases there is an immense resistance to very sudden changes of condition."

The second point of difference is that high-frequency currents pass chiefly along the "surface" of metallic conductors, whereas the continuous current flows equally in the deep and superficial parts. Thus hollow tubes conduct high-frequency currents equally as well as solid ones. On the other hand, when a saline fluid is substituted for the metal it is found that the solution is heated practically as much at the centre as at the periphery. As far as living tissue goes, there is a certain combination of these effects, and in practice we know from experience that these alternating currents do penetrate the deep structures of the parts lying between the electrodes.

### Application and Precautions.

Without going into further details it may be pointed out that the noticeable effect of passing the high-frequency current through a portion of the human body, a limb, for example, consists entirely in the warmth produced. If the pads are perfectly moist, flat, and carefully applied, and no local sparking is allowed to occur in the neighbourhood of the flesh, then, if the current is gradually turned on, there is at first no sensation at all. Later the heating effect is gradually observed. If, for example, the terminals are held in the hands, at first nothing is felt, but very soon a pleasant sensation of warmth is appreciated at the wrists. The feeling gradually increases, slowly spreading up the arms, and after ten to fifteen minutes the patient begins to feel warm all over the body and the pulse rate usually rises. Sometimes there is a fall of blood pressure, but the effect both on the pulse rate and on the blood pressure depends on various factors—the condition of the patient, his heart, blood-vessels, and kidneys. In any case these effects should only be produced deliberately in cases medically fit to stand them, and this is certainly one of the quarters from which danger may arise.

A second and perhaps more important consideration is the fact that, although the most important guide to the amount of current which may be safely taken by any particular case is the feeling of the patient, and particularly his sensibility to actual heat, nevertheless a note of warning must be

* Published by Cassell and Co., London, price 7s. 6d. net.

**Diathermy—(*continued*).**

sounded as regards the treatment of people suffering from trophic changes in the nerves giving rise to anæsthetic or semi-anæsthetic areas. Also cases of injury or division of the nerves supplying the part must be guarded against. In this instance not only is a skilled preliminary examination essential, but also, as the patients are unable to appreciate painful stimuli, and therefore do not complain of pain or discomfort from overheating, the greatest care is required in regulating the application. A host of other small risks may be commonly encountered in connection with these electrical treatments. Extremely careful application of the electrodes is necessary to prevent overheating and sparking locally. Inflammatory conditions associated with walled-in pus, venous thrombosis, advanced arterio-sclerosis (blood-pressure effects), all hæmorrhagic conditions, local alimentary activity (such as following a meal), and many other states, not all easily recognised by the untrained, form an absolute contra-indication to the treatment.

### Value in Treatment.

Our object is not, however, to belittle the value of diathermy in medical work, and one has but to read Dr. Saberton's book to realise the great opportunities for dealing with intractable conditions which the high-frequency current can provide. For clear and convincing details we must refer the reader to the little book which originally drew our attention. Otherwise we have sketched only the essential principles, and, simple though they are, would point out that, as with most electric work, there is an element of risk, and therefore very special training should be obtained by the medical man and expected in him by the patient before such modes of treatment are undertaken or accepted.

